# Kostmann’s disease or severe hereditary neutropenia—the man behind the syndrome

**DOI:** 10.1007/s00277-020-04142-y

**Published:** 2020-06-23

**Authors:** Lars Stenhammar, Leif Strömberg, Carl Gustaf Ljunggren

**Affiliations:** Department of Pediatrics and Department of Clinical and Experimental Medicine, Norrköping Hospital, Linköping University, SE-60182 Norrköping, Sweden

**Keywords:** Kostmann’s disease, Hereditary neutropenia, Rolf Kostmann

## Abstract

Seventy years ago, the Swedish pediatrician Rolf Kostmann (1909–1982) was the first to report on a previous unknown lethal hereditary neutropenia in infants, Kostmann’s disease. This essay presents the man behind the syndrome rather than focusing on the disease itself.

Few illustrious medical researchers have been granted the privilege of having their name associated with a syndrome or disease, one of these was the Swedish pediatrician Rolf Kostmann (1909–1982) (Fig. 1). He was the first to report on a previously unknown lethal hereditary neutropenia in infants, which he first named “hereditary reticulosis” [1] and later “infantile genetic agranulocytosis” [2]. Seventy years have now passed since his seminal paper was published in a Swedish journal [1]; a fact that probably explains why his pioneer work did not gain the attention it deserved. Since then, the disease has been well documented in the literature [3–7]. The enthralling history behind Kostmann’s discovery and a portrait of the fascinating person himself were described in his Swedish memoirs [8], soon to be translated into English. The lucid autobiography, richly illustrated with his own linoleum prints (Fig. 2), provides delightful and exciting reading with amusing anecdotes from his hard-working and thrilling life. The authors of the present essay had the privilege of working under Rolf Kostmann in the late 1960s and early 1970s [9]. By reporting some of our enduring memories, we wish to draw attention to the man behind the syndrome rather than focusing on the disease itself.

**Fig. 1:**
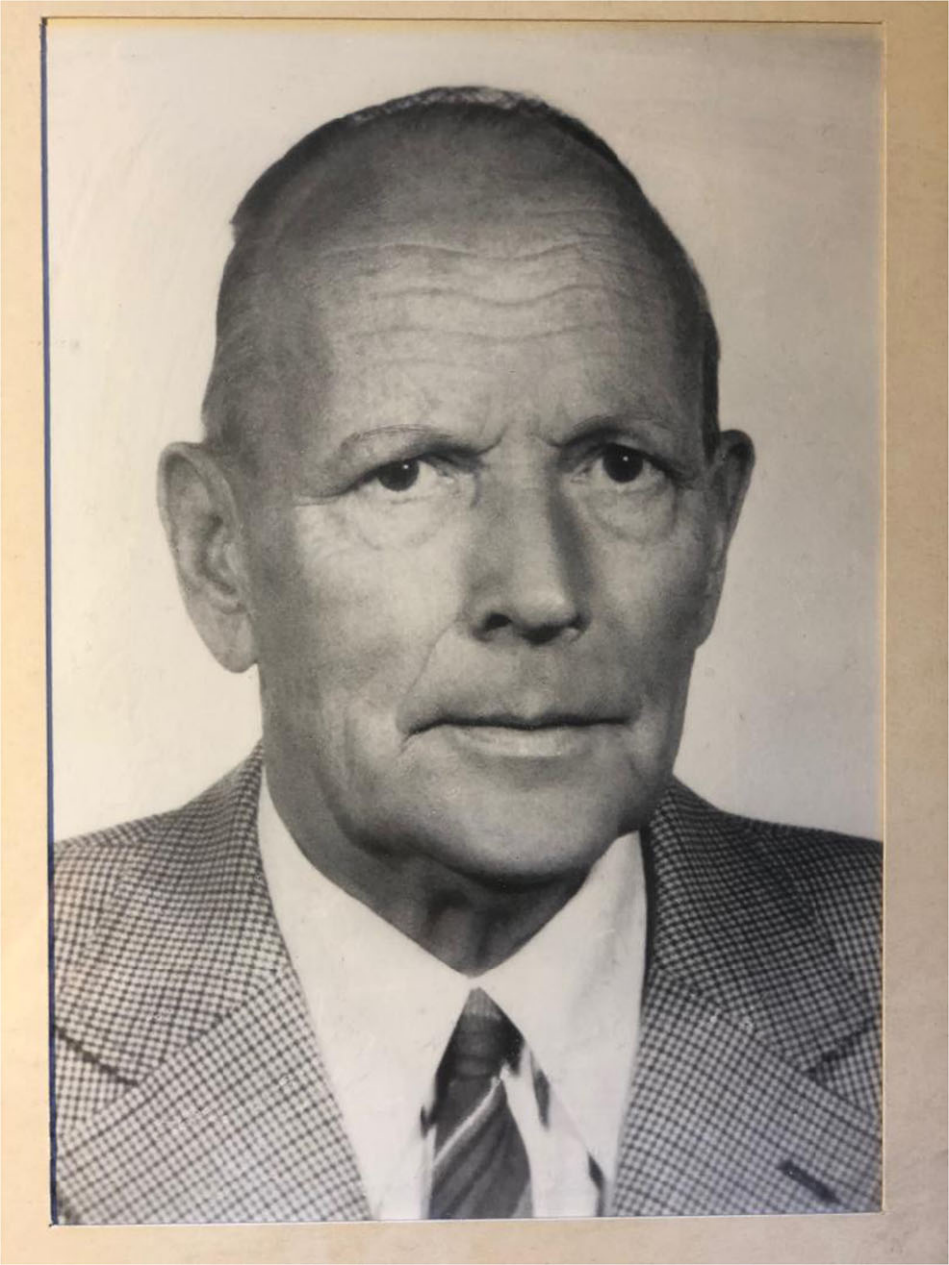
Rolf Kostmann (1909–1982). (Private photo)

**Fig. 2:**
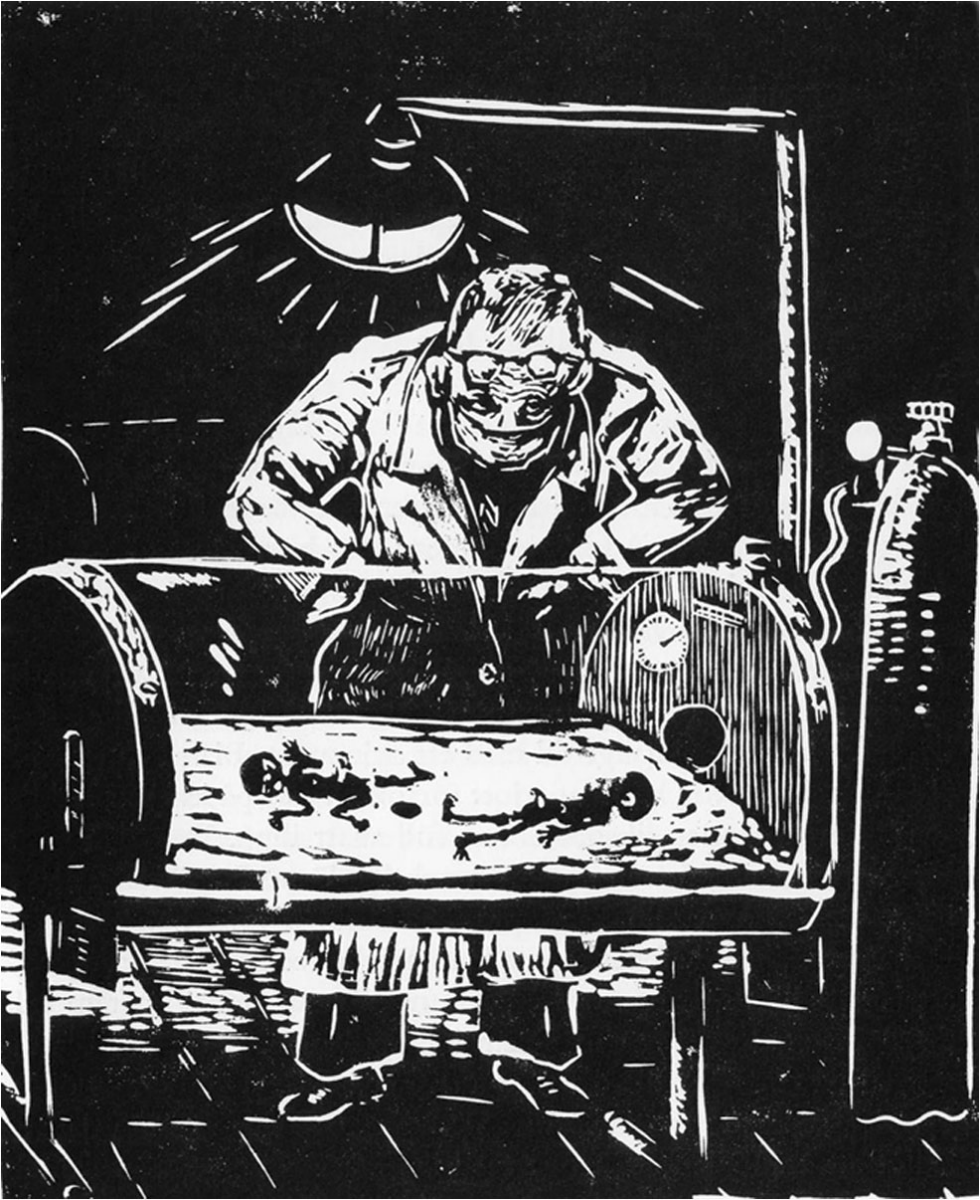
“Ordeals of the Night”. Linoleum printing by Rolf Kostmann

In 1941, Rolf Kostmann completed his pediatric training at one of the two university pediatric departments in Stockholm, Sweden. The following year, he was appointed half-time army surgeon in the Swedish military defense force, holding the rank of major and stationed in Boden, a small town in the most northerly county of Sweden. The other half of his occupation was spent as consultant pediatrician at the Army Hospital in Boden where he was commissioned to establish a pediatric clinic at the hospital. For several years, he was the only pediatrician to serve the vast but sparsely populated county. On top of that, he had the job of examining 3000 war refugee children from Finland. It is easy to see that the amount of work he carried out during World War II was huge. As time went by, he realized that he had to move from the “God-forsaken wilderness” in northern Sweden, not least to guarantee the future education of his children. To achieve this, he needed academic merits to have any chance of getting a position in the southern part of the country. However, he had no research background and no research project in mind.

Then, in March 1949, a 2-month-old girl was taken to the pediatric clinic by her mother, after having traveled 160 km along icy dirt tracks. The child had high fever, mastoiditis, otitis, several boils in her skin, and agranulocytosis but had not eaten neither a poison nor a drug. She had eight siblings, of whom four had died in infancy of a febrile condition, and three of them had also had boils. Furthermore, some infants on the father’s side of the family had suffered an illness with similar symptoms. This case was unique to Rolf Kostmann, who happened to have an interest in hematology. After further questioning of the mother, he suspected that this was a novel hereditary disturbance of the granulocytopoiesis process. He performed the microscopic leucocyte count himself. This became the starting point of his fascinating research. In short, Kostmann’s further research revealed that the origin of the disease was a mutation, which probably had occurred in the eighteenth century. He summarized that the child suffered from a recessively inherited disease with an absolute neutrophil count below 0.5 × 10^9^/L, criteria which are still referred to as Kostmann’s disease [1–5]. Later research work of clinicians and scientists determined the disease as an HAX1 mutation, which opened up the possibility of therapeutic interventions [6, 7].

In 1950, Rolf Kostmann fell ill with recurrent endogenous depression that cast a shadow over his and his family’s life. He was quite open about his illness and was one of the first patients in Sweden to be treated with electroconvulsive therapy. He often told us that without this he would never have survived. Two years later, he was appointed head of the Pediatric Clinic at Norrköping Central Hospital in south-east Sweden, the fourth largest city in the country at that time. Delayed by periods of depression, he finally and successfully defended his doctoral thesis in 1956, a thesis that included the results of 700 registered ancestral pathways [2].

Apart from being an excellent clinician and scientist, Rolf Kostmann was an extremely gifted person, a most entertaining and humorous speaker, and a skillful artist when it came to drawing and linoleum printing. An example of his technical skill and scientific curiosity was his construction of a reflecting telescope, enabling him to study the planets. Moreover, he was a good fencer and knew how to handle a rifle. Not without a degree of malicious pleasure, he told us how he gained the respect of the regimental commander during the war by winning a compulsory shooting competition against the rest of the officers. Many years later, in Norrköping, he put his art of fencing to work fighting duels with the chief orthopedic surgeon, also a dedicated fencer, during lunchtime breaks in the baby pram garage. To an outsider, this looked quite violent, but as far as we know, neither of the combatants suffered injury nor any of the patients or personnel. Rolf Kostmann showed that he was a man of action when one of his colleagues was life-threatened by a furious father who claimed his son had been mistreated, causing a wave of anxiety to spread amongst the personnel at the department. One day, the father was due to come to the ward. Prior to the morning round that day, Rolf Kostmann slightly opened his white coat to reveal an impressive knife hanging from his belt. With a quick movement, he drew the knife ready for attack shouting “Come on, you bastard!”. As luck would have it, the father never showed up. It should be apparent to the reader that Rolf Kostmann was a person who commanded respect and was willing to use force when necessary to defend himself or members of his staff. Furthermore, he often brought his big wolfhound Harras to work, guarding his office with growling that caused any unauthorized visitor to make a prompt retreat.

As departmental chief, Rolf Kostmann was candid, honest, straightforward, and supportive. He was humorous and inspired those around him with his enthusiasm. He encouraged us junior colleagues to start research projects and to design and build simple equipment for CPAP treatment or phototherapy for infants. As time progressed, the periods of depression that haunted and hampered him became more frequent and lingering. This was probably the main reason for his premature retirement in 1972 at the age of 63. However, he was then able to resume his previous research work with renewed strength, which resulted in two more publications [10, 11],

In 1982, just 73 years-old, he passed away after a short illness. We, former members of his staff, often talk about him with fond memories, remembering him as the outstanding pediatrician and warm and generous person he was. To us, he is truly unforgettable.
